# Exploring the key genes and pathways of side population cells in human osteosarcoma using gene expression array analysis

**DOI:** 10.1186/s13018-018-0860-8

**Published:** 2018-06-19

**Authors:** Yi-Ming Ren, Yuan-Hui Duan, Yun-Bo Sun, Tao Yang, Wen-Jun Zhao, Dong-Liang Zhang, Zheng-Wei Tian, Meng-Qiang Tian

**Affiliations:** Department of Joint and Sport Medicine, Tianjin Union Medical Center, Jieyuan Road 190, Hongqiao District, Tianjin, 300121 People’s Republic of China

**Keywords:** Osteosarcoma, Side population cells, Differentially expressed genes, Bioinformatics analysis

## Abstract

**Background:**

Human osteosarcoma (OS) is one of the most common primary bone sarcoma, because of early metastasis and few treatment strategies. It has been reported that the tumorigenicity and self-renewal capacity of side population (SP) cells play roles in human OS via regulating of target genes. This study aims to complement the differentially expressed genes (DEGs) that regulated between the SP cells and the non-SP cells from primary human OS and identify their functions and molecular pathways associated with OS.

**Methods:**

The gene expression profile GSE63390 was downloaded, and bioinformatics analysis was made.

**Results:**

One hundred forty-one DEGs totally were identified. Among them, 72 DEGs (51.06%) were overexpressed, and the remaining 69 DEGs (48.94%) were underexpressed. Gene ontology (GO) and pathway enrichment analysis of target genes were performed. We furthermore identified some relevant core genes using gene–gene interaction network analysis such as EIF4E, FAU, HSPD1, IL-6, and KISS1, which may have a relationship with the development process of OS. We also discovered that EIF4E/mTOR signaling pathway could be a potential research target for therapy and tumorigenesis of OS.

**Conclusion:**

This analysis provides a comprehensive understanding of the roles of DEGs coming from SP cells in the development of OS. However, these predictions need further experimental validation in future studies.

## Background

Osteosarcoma (OS), which is produced by mesenchymal cells, is the most common primary malignant tumor originating from bone tissues. OS occurs mainly in children and adolescents and accounts for 8.9% of cancer-related diseases which lead to death [1, 2]. Although new therapies of neoadjuvant chemotherapy and surgery have contributed greatly to OS treatment, the 5-year survival rate of OS is difficult to exceed 60–65% [3, 4]. To sum up, the early diagnosis and effective treatment of OS are the breakthrough point of OS research and clinical application, and exploring the pathogenesis, development, and metastasis of OS is the key. Although a large number of studies have been made on OS at molecular and cellular levels, the mechanisms of OS formation and metastasis have not been fully elucidated.

Side population (SP) cells are a group of special cells, which were found when Hoechst and flow cytometry are used to separate hematopoietic stem cells and progenitor cells. SP cells are widely distributed in a variety of adult tissues, embryos, and some tumor cell lines [5]. They not only have self-renewal and multipotential differentiation potential, but also have unique phenotypic markers and biological characteristics of stem cells, whose characteristics are very similar to those of tumor stem cells [6, 7]. SP cells help maintain the tumorigenic potential of some tumor cell lines [8–10]. Ho et al. reported that SP cells were enriched in tumor-initiating capability compared with non-SP cells by nonobese diabetic/severe combined immunodeficiency xenograft experiments. Matrigel invasion assay showed that SP cells also have higher potential for invasiveness. Human telomerase reverse transcriptase expression was higher in the SP cells, suggesting that this fraction may represent a reservoir with unlimited proliferative potential for generating cancer cells [11]. Chiba et al. hold that a minority population of SP cells detected in hepatocellular carcinoma cells possessed extreme tumorigenic potential and provided heterogeneity to the cancer stem cell system characterized by distinct hierarchy [12]. Wang et al. observed a strong tumorigenesis ability of SP cells from HeLa cell line following in vivo transplantation into 5- to 6-week-old female Balb/c mice [13]. These findings indicate that SP cells is an enriched source of tumor-initiating cells with stem cell properties and may be an important target for effective therapy and a useful tool to investigate the tumorigenic process.

Interestingly, SP cells are present in primary mesenchymal neoplasms, including primary OS [14]. Here, we downloaded the gene expression profile GSE63390 from the Gene Expression Omnibus (GEO) database and made bioinformatics analysis to investigate differentially expressed genes (DEGs) that regulated between the SP cells and the non-SP cells from primary human OS. By doing this, we hope that the key target genes and pathways involved in the carcinogenesis and progression of human OS could be identified and existing molecular mechanisms could be revealed.

## Methods

### Gene expression microarray data

The gene expression profile GSE63390 was downloaded from the Gene Expression Omnibus (GEO, www.ncbi.nlm.nih.gov/geo/). GSE63390 was based on Illumina Human HT-12 V4.0 expression beadchip GPL10558 platform. The GSE63390 dataset contained three samples, including three SP cell samples and three non-SP cell samples.

### DEGs in SP cells and non-SP cells

The raw data files used for the analysis included TXT files (Illumina platform). The analysis was carried out using GEO2R, which can perform comparisons on original submitter-supplied processed data tables using the GEO query and limma R packages from Bioconductor project. The *P* value < 0.05 and log fold change (FC) > 1.0 or log FC < − 1.0 were used as the cut-off criteria. The DEGs with statistical significance between the SP cells and non-SP cells were selected and identified.

### GO and KEGG analysis of DEGs

Target genes list were submitted to the Cytoscape software version 3.4.0 (www.cytoscape.org) and ClueGO version 2.33 to identify overrepresented GO categories and pathway categories. Gene Ontology (GO) analysis was used to predict the potential functions of the DEGs in biological process (BP), molecular function (MF), and cellular component (CC). The Kyoto Encyclopedia of Genes and Genomes (KEGG, http://www.genome.jp/) is a knowledge base for systematic analysis of gene functions, linking genomic information with higher-level systemic functions. Finally, the overrepresented pathway categories with a *P* value < 0.05 were considered statistically significant using KEGG pathway enrichment analysis.

### Gene interaction network construction

A large number of DEGs we obtained may be human OS-associated genes, and it is suggested that these DEGs in SP cells may participate in the progression of human OS. Firstly, DEGs list was submitted to the Search Tool for the Retrieval of Interacting Genes (STRING) database (http://www.string-db.org/) and an interaction network chart with a combined score > 0.4 was saved and exported. Subsequently, the interaction regulatory network of human OS-associated genes was visualized using Cytoscape software version 3.4.0. The distribution of core genes in the interaction network was made by NetworkAnalyzer in Cytoscape. Then, the plugin Molecular Complex Detection (MCODE) was applied to screen the modules of the gene interaction network in Cytoscape.

## Results

### Identification of DEGs

The gene expression profile GSE63390 was downloaded from the GEO, and the GEO2R method was used to identify DEGs in SP cells compared with non-SP cells. *P* value < 0.05, log FC > 1.0, or log FC < − 1.0 were used as the cut-off criteria. After analyzing, differentially expression gene profiles were obtained. Totally, 141 DEGs were identified including 72 upregulated DEGs and 69 downregulated DEGs screened in SP cells of human OS compared with non-SP cells. Parts of DEGs were listed in Table 1.

**Table 1:** The top 10 regulated DEGs in OS SP cells with *P* value < 0.05

ID	*P* value	logFC	Gene symbol
Upregulated			
ILMN_2184250	6.45E−05	3.0817572	SERPINB9
ILMN_1713706	4.08E−02	3.08089766	ZNF786
ILMN_2260756	4.98E−02	2.76569527	GSDMB
ILMN_2189870	3.65E−03	2.52175439	FCF1
ILMN_2078724	2.68E−02	2.46037207	APOPT1
ILMN_1681490	3.27E−02	2.34502196	ZNF568
ILMN_1809957	1.68E−03	2.32352121	AP2S1
ILMN_1812392	1.46E−02	2.28124068	TMSB10
ILMN_2246548	3.62E−02	2.24405561	GSTTP2
ILMN_2053178	0.96444	2.21210244	ACTG1
Downregulated			
ILMN_1789196	2.84E−03	− 2.95354379	TPM2
ILMN_1672496	2.45E−04	− 2.53681489	DNAJA1
ILMN_1755733	7.92E−03	− 2.32101519	RPLP2
ILMN_1690494	3.37E−03	− 2.30094686	RPL6
ILMN_1686367	1.42E−02	− 2.28782734	HSPA8
ILMN_1728870	6.65E−03	− 2.24915587	DDX3X
ILMN_2230624	2.93E−03	− 2.22730708	RPL18
ILMN_1666385	5.79E−03	− 2.18496943	CALM3
ILMN_2378868	3.56E−02	− 2.17417408	SRSF5
ILMN_2139943	3.81E−03	− 2.15657539	RPS3A

*OS* osteosarcoma, *DEGs* differentially expressed genes, *SP* side population, *FC* fold change

### GO term enrichment analysis of DEGs

**Fig. 1:**
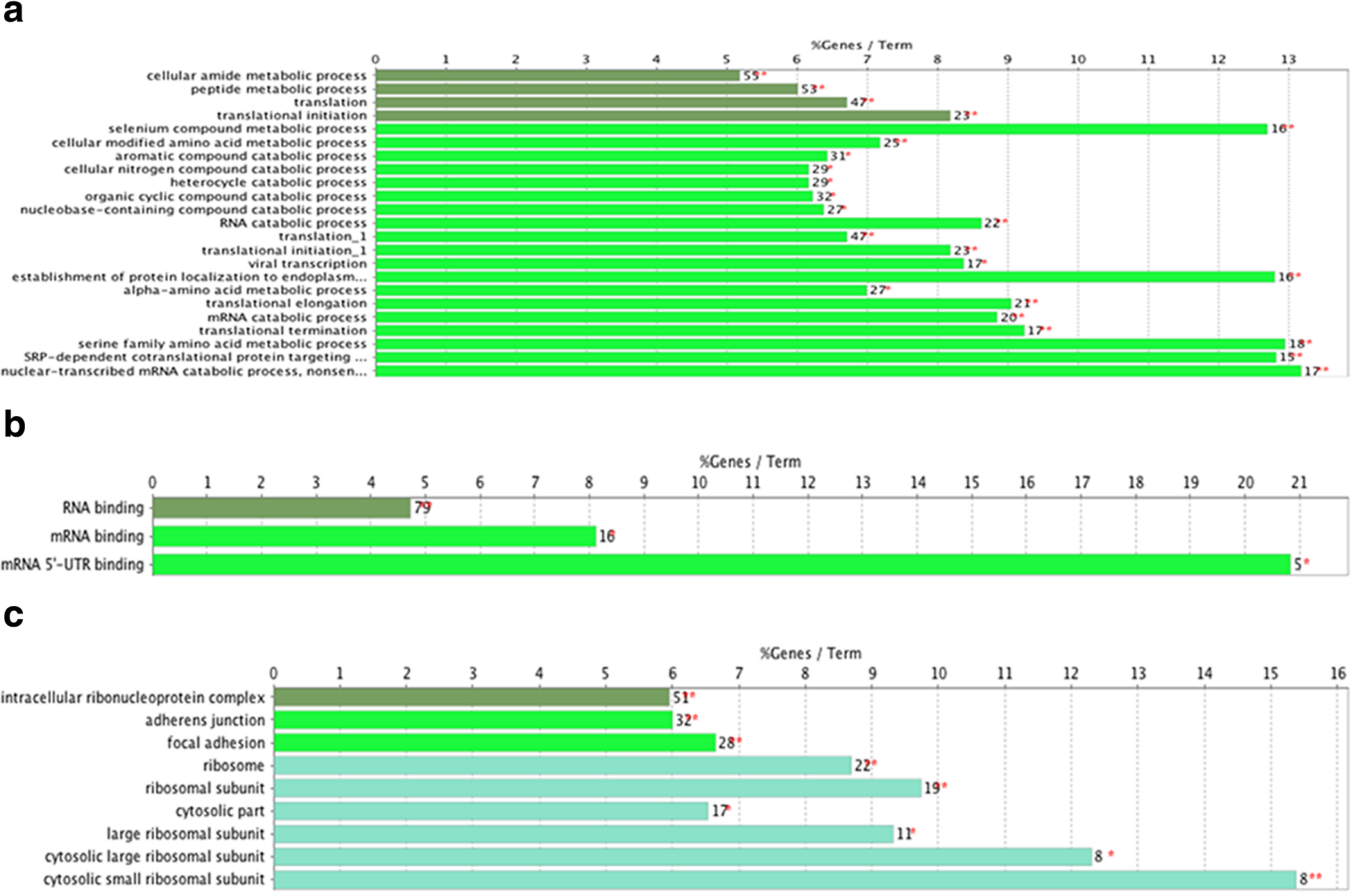
Gene ontology (GO) enrichment analysis of biological processes (**a**), molecular functions (**b**), and cellular components (**c**). The red star in GO terms means term *P* value < 0.05, and the double red stars in GO terms mean term *P* value < 0.01

Functional annotation of the 141 DEGs was clarified using the Cytoscape software online tool. GO analysis indicated that these DEGs were significantly enriched in cellular amide metabolic process, peptide metabolic process, translation, translational initiation, selenium compound metabolic process, cellular modified amino acid metabolic process, aromatic compound catabolic process, cellular nitrogen compound catabolic process, heterocycle catabolic process, organic cyclic compound catabolic process, nucleobase-containing compound catabolic process, RNA catabolic process, viral transcription, establishment of protein, localization to endoplasmic reticulum, alpha-amino acid metabolic process, translational elongation, mRNA catabolic process, translational termination, serine family amino acid metabolic process, SRP-dependent cotranslational protein targeting to membrane, nuclear-transcribed mRNA catabolic process, nonsense-mediated decay, and other biological processes (Fig. 1). For MF, the DEGs were enriched in RNA binding, mRNA binding, mRNA 5′-UTR binding and others. In addition, GO CC analysis also showed that the DEGs were significantly enriched in intracellular ribonucleoprotein complex, adherens junction, focal adhesion, ribosome, ribosomal subunit, cytosolic part, large ribosomal subunit, cytosolic large ribosomal subunit, cytosolic small ribosomal subunit, and others.

### KEGG pathway analysis of DEGs

**Fig. 2:**
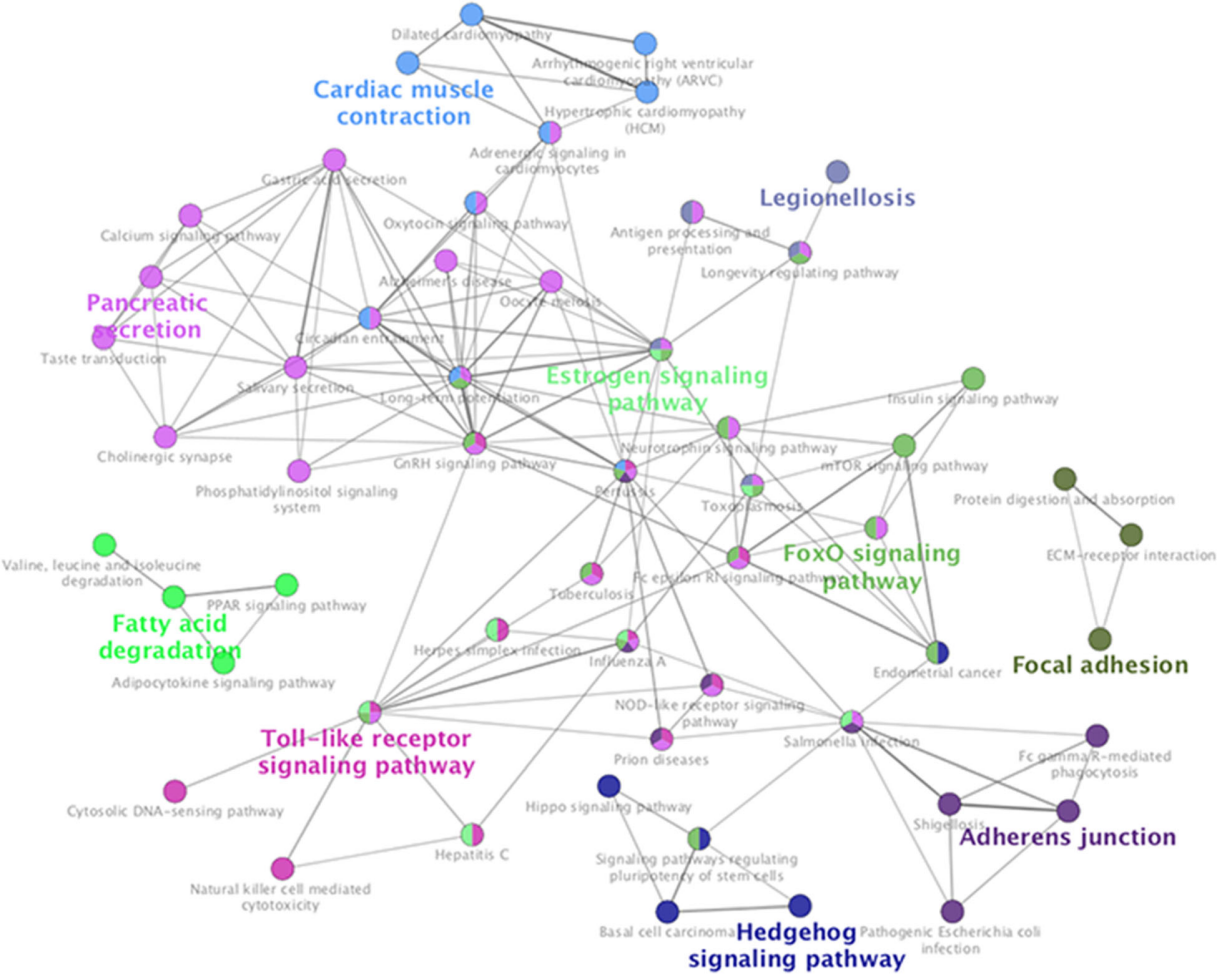
Kyoto Encyclopedia of Genes and Genomes (KEGG) pathway analysis of differentially expressed genes (DEGs). The different node colors mean different pathways, and the closer the colors are, the closer the function clustering of pathways are

The result of KEGG pathway analysis revealed that target genes were enriched in estrogen signaling pathway, hippo signaling pathway, adherens junction, NOD-like receptor signaling pathway, apelin signaling pathway, ECM–receptor interaction, Toll-like receptor signaling pathway, mTOR signaling pathway, FoxO signaling pathway, cell adhesion molecules (CAMs) and hedgehog signaling pathway, and others. These key pathways were showed in Fig. 2. Fifty-five nodes and 163 edges could be discovered in this network. FoxO signaling pathway and mTOR signaling pathway clustered together. Estrogen signaling pathway and hippo signaling pathway clustered together. Besides, these core pathways and their associated genes found were summarized in Table 2. The first-ranking estrogen signaling pathway had the 6.12% associated genes, which included CALM3, CALML4, HSPA1L, HSPA8, ITPR3, and MAPK3. The second-placed hippo signaling pathway had the 5.84% associated genes, which included ACTG1, APC2, BIRC2, BMP2, FRMD6, LLGL2, RASSF6, SOX2, and WNT8A.

**Table 2:** Core pathways and their associated genes found

GOID	GOTerm	Term *P* value	% associated genes	Associated genes found
GO:0004915	Estrogen signaling pathway	130.0E−3	6.12	[CALM3, CALML4, HSPA1L, HSPA8, ITPR3, MAPK3]
GO:0004390	Hippo signaling pathway	62.0E−3	5.84	[ACTG1, APC2, BIRC2, BMP2, FRMD6, LLGL2, RASSF6, SOX2, WNT8A]
GO:0004520	Adherens junction	290.0E−3	5.56	[ACTG1, MAPK3, PTPRM, WAS]
GO:0004657	IL-17 signaling pathway	220.0E−3	5.38	[IL6, MAPK15, MAPK3, S100A8, S100A9]
GO:0004621	NOD-like receptor signaling pathway	110.0E−3	5.29	[BIRC2, ERBIN, IFNA1, IFNAR2, IL6, ITPR3, MAPK3, RNASEL, TP53BP1]
GO:0004210	Apoptosis	210.0E−3	5.07	[ACTG1, BIRC2, ITPR3, LMNA, MAPK3, PDPK1, TUBA3D]
GO:0004371	Apelin signaling pathway	210.0E−3	5.07	[CALM3, CALML4, GNG11, ITPR3, MAPK3, PRKAG2, RYR2]
GO:0004722	Neurotrophin signaling pathway	270.0E−3	5.04	[CALM3, CALML4, IRAK2, MAGED1, MAPK3, PDPK1]
GO:0004020	Calcium signaling pathway	190.0E−3	4.95	[CALM3, CALML4, CHRM3, ITPR3, P2RX2, PTGER3, RYR2, SLC25A4, STIM2]
GO:0004512	ECM–receptor interaction	330.0E−3	4.88	[COL1A2, COL2A1, COL6A1, HSPG2]
GO:0004620	Toll-like receptor signaling pathway	380.0E−3	4.81	[IFNA1, IFNAR2, IL6, MAP2K3, MAPK3]
GO:0004150	mTOR signaling pathway	340.0E−3	4.61	[ATP6V1B2, DEPDC5, EIF4E, MAPK3, PDPK1, PRR5, WNT8A]
GO:0004068	FoxO signaling pathway	310.0E−3	4.55	[BCL6, CCNG2, IL6, MAPK3, PDPK1, PRKAG2]
GO:0004510	Focal adhesion	290.0E−3	4.52	[ACTG1, BCAR1, BIRC2, COL1A2, COL2A1, COL6A1, MAPK3, PARVB, PDPK1]
GO:0004514	Cell adhesion molecules (CAMs)	460.0E−3	4.14	[CADM1, CLDN5, NCAM2, PTPRC, PTPRM, SELP]

### Interaction network of DEGs and core genes in the interaction network

**Fig. 3:**
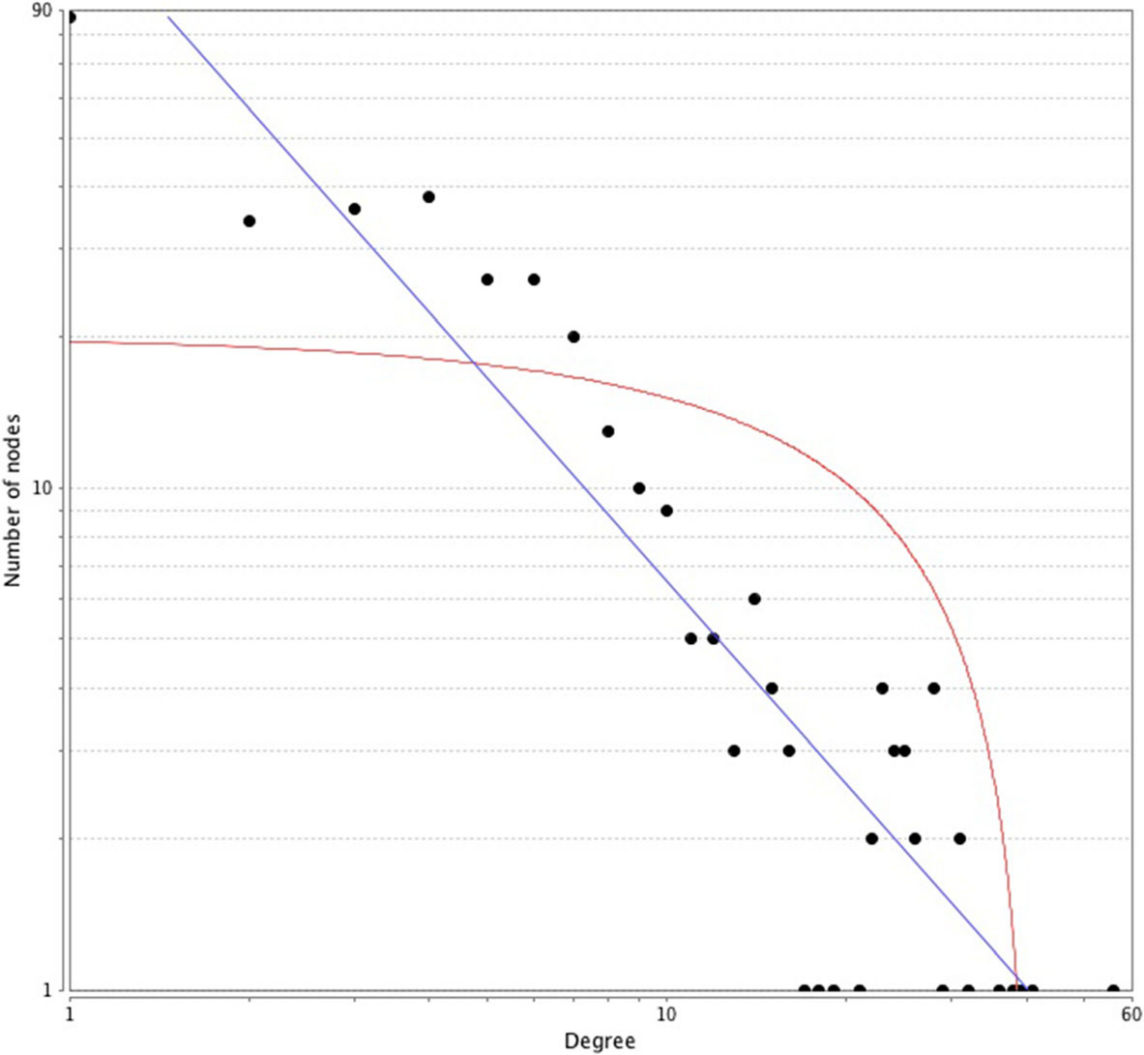
The distribution of core genes in the interaction network. The black node means the core gene. The red line means the fitted line, and the blue line means the power law. The correlation between the data points and corresponding points on the line is approximately 0.932. The R-squared value is 0.846, giving a relatively high confidence that the underlying model is indeed linear

**Fig. 4:**
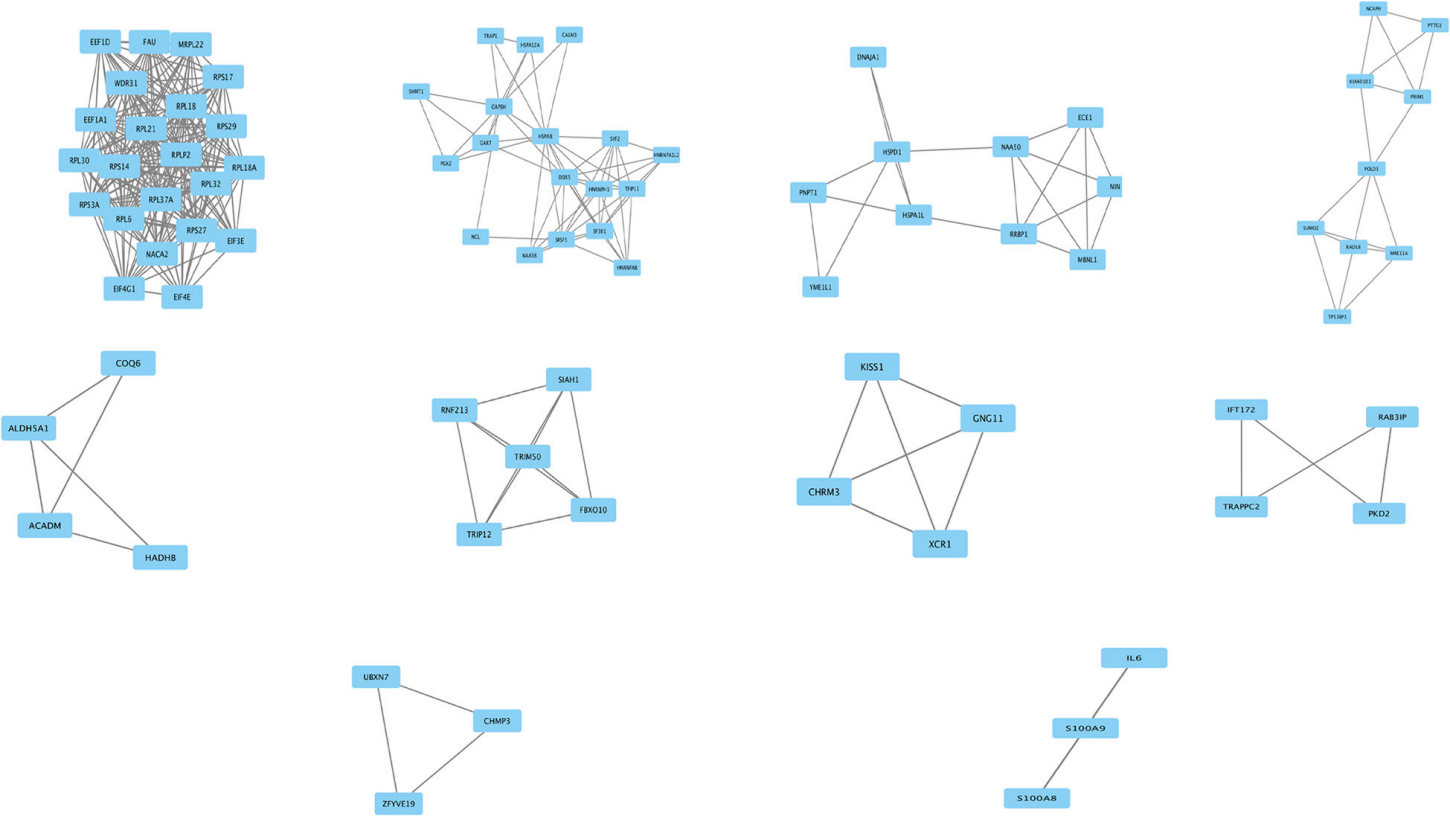
The top 10 modules from the gene–gene interaction network. The squares represent the differentially expressed genes (DEGs) in modules, and the lines show the interaction between the DEGs

Based on the information in the STRING database, the gene interaction network contained 542 nodes and 1163 edges. The nodes indicated the DEGs, and the edges indicated the interactions between the DEGs. NetworkAnalyzer in Cytoscape software was used to analysis these genes, and core genes were ranked according to the predicted scores. The top 10 high-degree hub nodes included glyceraldehyde-3-phosphate dehydrogenase (GAPDH), phosphoribosylglycinamide formyltransferase, phosphoribosylglycinamide synthetase, phosphoribosylaminoimidazole synthetase (GART), ubiquitin-like and ribosomal protein S30 fusion (FAU), heat shock protein family A member 8 (HSPA8), eukaryotic translation elongation factor 1 alpha 1 (EEF1A1), ribosomal protein S3A (RPS3A), eukaryotic translation initiation factor 4E (EIF4E), mitogen-activated protein kinase 3 (MAPK3), interleukin 6 (IL6), and ribosomal protein L6 (RPL6). Among these genes, GAPDH showed the highest node degree, which was 56. The core genes and their corresponding degree were shown in Table 3. The distribution of core genes in the interaction network was revealed in Fig. 3. The correlation between the data points and corresponding points on the line is approximately 0.932. The R-squared value is 0.846, giving a relatively high confidence that the underlying model is indeed linear. Then, we used MCODE to screen the modules of the gene interaction network, and 10 modules were showed in Fig. 4.

**Table 3:** The core genes and their corresponding degree

Gene	Degree	Gene	Degree	Gene	Degree	Gene	Degree
GAPDH	56	RPS3A	32	EIF3b	28	RPL18	25
GART	41	EIF4E	31	DDX5	28	CALM3	25
FAU	39	MAPK3	31	HSPD1	28	ACTG1	25
HSPA8	38	IL6	29	RPS29	26	RPS27	24
EEF1A1	36	RPL6	28	RPL18A	26	RPL32	24

**Fig. 5:**
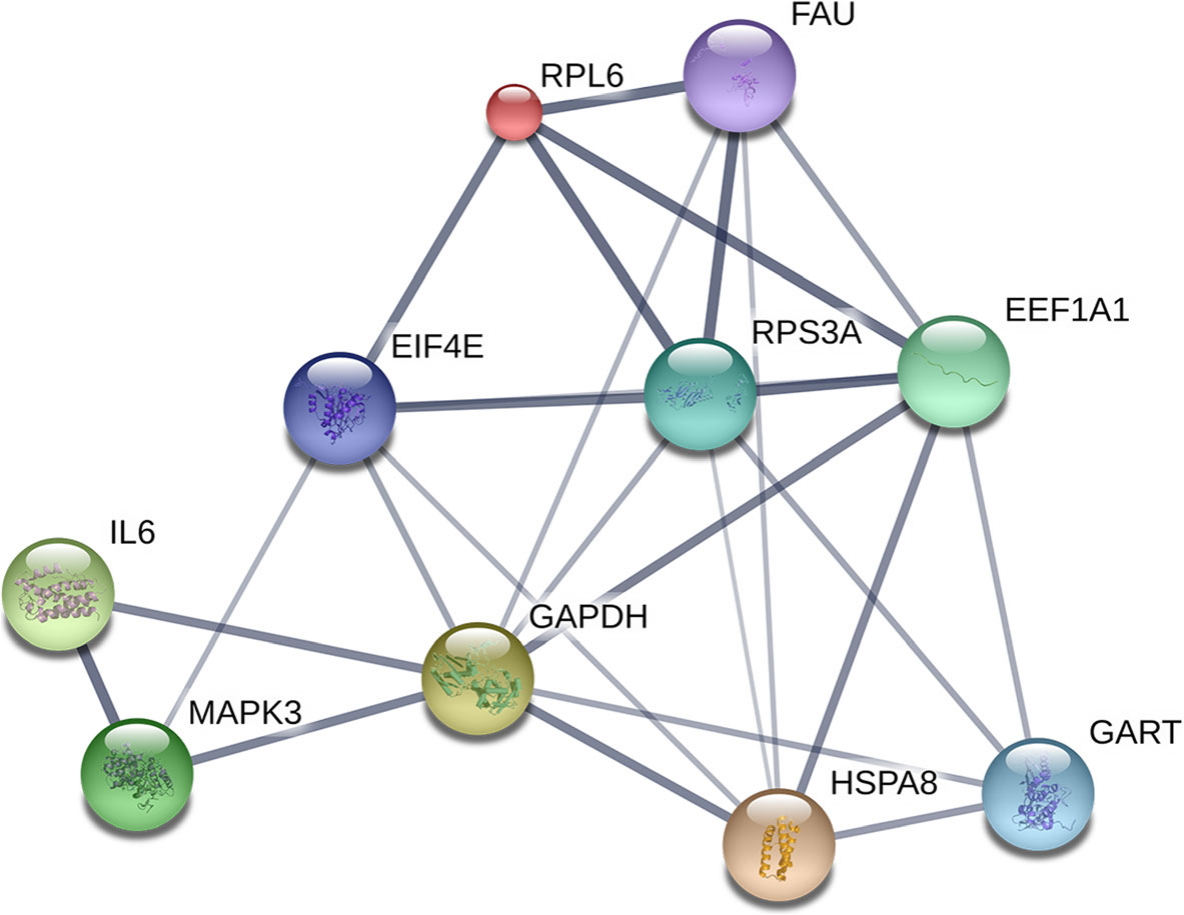
The interaction network of the top 10 core genes. The nodes indicated the top core genes, and the edges indicated the interactions between the core genes

The score of top 1 module including FAU and EIF4E was 19.81, which had 22 nodes and 208 edges. The score of top 2 module including GAPDH and GART was 6.824, which had 18 nodes and 58 edges. The score of top 3 module including FBXO10, RNF213, SIAH1, TRIM50, and TRIP12 was 5, which had 5 nodes and 10 edges. Lastly, the interaction network of the top 10 high-degree hub nodes (core genes) was made by STRING database in Fig. 5. GAPDH, GART, FAU, HSPA8, EEF1A1, RPS3A, EIF4E, MAPK3, IL6, and RPL6, which regulate 8, 4, 5, 6, 6, 6, 5, 3, 2, and 4 targets, respectively, showed the good connectivity.

## Discussion

OS is the most common primary bone sarcoma [15–19]. Previous studies discovered that sarcomas contain a small subpopulation of tumor-propagating cells (TPCs) such as SP cells, characterized by enhanced tumorigenicity and self-renewal capacity [20–22]. Self-renewal is a defining characteristic of these cells and is associated with tumor recurrence [23, 24]. The inhibition of self-renewal in OS SP cells may offer valuable targets of therapy and tumorigenesis mechanisms. In the present study, the gene expression profile of GSE63390 was downloaded and a bioinformatics analysis was performed. The results showed that there were 141 DEGs in SP cells compared with non-SP cells of human OS. Furthermore, GO and KEGG pathway and gene–gene interaction network analysis were performed to obtain the biomarkers or the major genes related to cytogenetic pathways to OS tumorigenesis.

In order to disclose the underlying molecular mechanisms between SP cells and human OS, we characterized the possible GO functional terms and signaling pathways of DEGs. Considering the results of GO function analysis, we linked the DEGs with mRNA catabolic process and cellular modified amino acid metabolic process, which are probably very important for the development process of human OS. As previous articles reported, our KEGG pathway analysis showed that hippo signaling pathway, mTOR signaling pathway, hedgehog signaling pathway, and others were among the most relevant pathways for OS. Zhou et al. found that the correlation between the mTOR/p70S6K signal transduction pathway in human OS and patients’ prognosis, and the overexpression of mTOR and p70S6K, is well correlated with tumor metastasis pattern, which might be an important mechanism responsible for the survival and proliferation of OS cells [25]. Wang et al. identified that hippo/YAP signaling pathway not only is involved in tumorigenesis, but also hippo/YAP signaling pathway induces OS chemoresistance [26]. Chai et al. deemed that the oncogenic activities in OS are mediated by TED1 through hippo–YAP1 signaling [27]. Cheng et al. highlighted a new discovery that CNOT1–LMNA–Hedgehog signaling pathway axis exerts an oncogenic role in OS progression, which could be a potential target for gene therapy [28]. Emerging data suggested that interference with hedgehog signaling signal transduction by inhibitors may reduce OS cell proliferation and tumor growth, thereby preventing osteosarcomagenesis [29]. All these signaling pathways may play important roles in molecular mechanism of development process between SP cells and human OS.

Also of note is that there were numerous evidences for our DEGs of SP cells, which have proven to play important roles during OS tumorigenesis. The STRING database revealed top 20 high-degree hub nodes of DEGs including GAPDH, GART, FAU, HSPA8, EEF1A1, RPS3A, EIF4E, MAPK3, IL6, RPL6, eukaryotic translation initiation factor 3 beta (EIF3b), DEAD-box helicase 5 (DDX5), heat shock protein family D member 1 (HSPD1), ribosomal protein S29 (RPS29), ribosomal protein L18a (RPL18A), ribosomal protein L18 (RPL18), calmodulin 3 (CALM3), actin gamma 1 (ACTG1), ribosomal protein S27 (RPS27), and ribosomal protein L32 (RPL32). Furthermore, we analyzed the gene interaction network and top 10 modules using MCODE and found that ACTG1, eukaryotic translation initiation factor 3 subunit E (EIF3E), EIF4E, FAU, HSPD1, IL-6, KiSS-1 metastasis-suppressor (KISS1), PRIM1, pituitary tumor-transforming 1 (PTTG1), PRL32, S100 calcium-binding protein A8 (S100A8), S100 calcium-binding protein A9 (S100A9), serine hydroxymethyltransferase 1 (SHMT1), and TNF receptor-associated protein 1 (TRAP1) were the core interaction genes, which may be potential therapeutic targets for OS. Parts of them were in accord with STRING database results. Ajiro et al. found that serine/arginine-rich splicing factor 3 (SRSF3) regulates the expression of DDX5 in human OS U2OS cells [30]. By participating in the transcriptional regulation of ribosomal protein L34 (RPL34) which plays an important role in the proliferation of OS cells, MYC interacts with the subunits of EIF3 and probably involves the translational control of growth-promoting proteins [31]. EIF3, a multi-subunit complex, plays a critical role in translation initiation. Expression levels of EIF3 subunits are elevated or decreased in various cancers, suggesting a role for EIF3 in tumorigenesis [32]. Choi et al. confirmed that EIF3b silencing could completely suppress cell growth in multiple OS cell lines [33]. Osborne et al. also discovered that EIF4E is uniformly expressed in OS patient samples and it could be a relevant protein biomarker in OS [34]. Rossman et al. found that overexpressing FAU itself is able to transform human osteogenic sarcoma cells to anchorage independence and make them easy to proliferate [35]. Liang et al. proved that the expression of HSPD1 was high in OS tissues and cells; moreover, targeted inhibition of this gene could inhibit the proliferation of the tumor [36]. Zhang et al. indicated that the decreased expression of KISS1 is correlated with distant metastasis of OS, and KISS1 may function as a tumor suppressor in OS cells through inhibition of the MAPK pathway [37]. EEF1A1 is overexpressed in OS cell lines, and siRNA treatment against EEF1A1 produces a chemosensitization toward methotrexate, which showed that this gene is a potential therapeutic target of OS [38]. Through ASK1/p38/AP-1 signal pathway, IL-6 occurs, which in turn results in the activations of vascular endothelial growth factor (VEGF) expression and contributing the angiogenesis of human OS cells [39]. In addition, the ILK/Akt/AP-1 pathway is activated after IL-6 treatment, and IL-6 induces expression of ICAM-1 and migration activity of human OS cells [40]. Yotov et al. proposed that PRIM1 is a major target of 12q13 amplifications, playing an essential role in tumorigenesis of human OS [41]. PTTG1 siRNA markedly downregulates the expression of PTTGl protein in OS cells, leading to obvious inhibition of cell proliferation, alters cell cycle distribution, and reduces ability of invasion of OS cells [42]. Tsai et al. deemed that expression stability of RPL32 is high in OS samples, and this gene could be a potential target [43]. In Montesano’s study, the anti-apoptotic role of TRAP1 is confirmed in Saos-2 OS cells, which suggested that increased expression of this gene could make diethylmaleate-adapted and chemoresistant cells evade toxic effects of oxidants and anticancer drugs [44]. Endo-Munoz et al. proved downregulation of S100A8 between chemo-naive OS biopsies and non-malignant bone biopsies, highlighting their potential as therapeutic targets for OS [45]. Cheng et al. confirmed that through inactivating MAPK and NF-κB signaling pathways, downregulation of S100A9 could inhibit OS cell growth [46]. Besides, Both et al. concluded that some genes, including SHMT1, are candidate oncogenes in 17p11.2–p12 of importance in OS tumorigenesis [47]. Taken together, all these core genes discovered in OS SP cells by bioinformatics enrichment analysis and gene interaction network analysis may increase or decrease tumorigenicity and self-renewal capacity of OS SP cells; further, these changed SP cells could result in development process of human OS.

Some gene and pathway interaction relationship predicted in our study has been reported in previous researches. Oncogenic activation of mTOR signaling significantly contributes to the progression of different types of cancers including OS. EIF4E is one of the downstream effectors of mTOR. Activated mTOR contributes to OS cellular transformation and poor cancer prognosis via targeting the downstream effectors such as EIF4E [48]. In addition, our results of core pathways and their associated genes found also confirmed the relationship of EIF4E and mTOR signaling pathway. Therefore, EIF4E/mTOR signaling pathway could be a potential research target for therapy and tumorigenesis of OS.

Lastly, there are several limitations of this study. It is acknowledged that predicting key genes merely by means of bioinformatics is not sufficient, and further molecular biological experiments such as the use of gene transfection/knockdown and quantitative real-time polymerase chain reaction are needed to confirm these results.

## Conclusion

In summary, 141 DEGs were identified including 72 upregulated DEGs and 69 downregulated DEGs screened in SP cells compared with non-SP cells. GO and KEGG pathway analysis provided a series of related key genes and pathways to contribute to the understanding of the molecular mechanisms between SP cells and human OS, thus yielding clues to speculate the EIF4E/mTOR signaling pathway is highly correlated with the development process of OS. Furthermore, these predictions need further experimental validation in future studies.

## Abbreviations

ACTG1: Actin gamma 1
BP: Biological process
CALM3: Calmodulin 3
CAMs: Cell adhesion molecules
CC: Cellular component
DDX5: DEAD-box helicase 5
DEG: Differentially expressed gene
EEF: Eukaryotic translation elongation factor
EIF3b: Eukaryotic translation initiation factor 3 beta
FAU: Ubiquitin-like and ribosomal protein S30 fusion
FC: Fold change
GAPDH: Glyceraldehyde-3-phosphate dehydrogenase
GART: Phosphoribosylglycinamide formyltransferase, phosphoribosylglycinamide synthetase, phosphoribosylaminoimidazole synthetase
GEO: Gene Expression Omnibus database
GO: Gene Ontology
HSPA8: Heat shock protein family A member 8
HSPD1: Heat shock protein family D member 1
IL6: Interleukin 6
KEGG: Kyoto Encyclopedia of Genes and Genomes
KISS1: KiSS-1 metastasis-suppressor
MAPK3: Mitogen-activated protein kinase 3
MCODE: Molecular complex detection
MF: Molecular function
OS: Osteosarcoma
PTTG1: Pituitary tumor-transforming 1
RPL6: Ribosomal protein L6
RPS: Ribosomal protein
S100A8: S100 calcium-binding protein A8
SHMT1: Serine hydroxymethyltransferase 1
SP: Side population
SRSF3: Serine/arginine-rich splicing factor 3
STRING: Search Tool for the Retrieval of Interacting Genes
TPC: Tumor-propagating cell
TRAP1: TNF receptor associated protein 1
VEGF: Vascular endothelial growth factor
